# Selection and drift influence genetic differentiation of insular Canada lynx (*Lynx canadensis*) on Newfoundland and Cape Breton Island

**DOI:** 10.1002/ece3.2945

**Published:** 2017-04-09

**Authors:** Melanie B. Prentice, Jeff Bowman, Kamal Khidas, Erin L. Koen, Jeffrey R. Row, Dennis L. Murray, Paul J. Wilson

**Affiliations:** ^1^ Department of Environmental and Life SciencesTrent UniversityPeterboroughONCanada; ^2^ Wildlife Research and Monitoring SectionOntario Ministry of Natural Resources and ForestryPeterboroughONCanada; ^3^ Vertebrate ZoologyCanadian Museum of NatureOttawaONCanada; ^4^ Biology DepartmentTrent UniversityPeterboroughONCanada; ^5^ Department of Environment and Resource StudiesUniversity of WaterlooWaterlooONCanada

**Keywords:** adaptation, Canada lynx, genetic drift, IGF‐1, island rule, natural selection

## Abstract

Island populations have long been important for understanding the dynamics and mechanisms of evolution in natural systems. While genetic drift is often strong on islands due to founder events and population bottlenecks, the strength of selection can also be strong enough to counteract the effects of drift. Here, we used several analyses to identify the roles of genetic drift and selection on genetic differentiation and diversity of Canada lynx (*Lynx canadensis*) across eastern Canada, including the islands of Cape Breton and Newfoundland. Specifically, we assessed whether we could identify a genetic component to the observed morphological differentiation that has been reported across insular and mainland lynx. We used a dinucleotide repeat within the promoter region of a functional gene that has been linked to mammalian body size, insulin‐like growth factor‐1 (*IGF‐1*). We found high genetic differentiation at neutral molecular markers but convergence of allele frequencies at the *IGF‐1* locus. Thus, we showed that while genetic drift has influenced the observed genetic structure of lynx at neutral molecular markers, natural selection has also played a role in the observed patterns of genetic diversity at the *IGF‐1* locus of insular lynx.

## 1 Introduction

Island populations are extreme examples of genetic divergence caused by geographical and landscape barriers to gene flow, where large bodies of water inhibit the immigration of new individuals from the mainland (MacArthur & Wilson, 1967). Such insular populations are often characterized by small population sizes, which are largely the result of founder events and recurrent population bottlenecks, leading to the low‐genetic diversity of island‐dwelling populations via genetic drift (Frankham, 1997; Nei, Maruyama, & Chakraborty, 1975). Ultimately, small population sizes can result in an increased probability of extinction through demographic stochasticity and inbreeding depression, in addition to limiting the adaptability of island populations to environmental change (Hedrick & Kalinowski, 2000; Reed & Frankham, 2003). For example, a decrease in genetic diversity due to genetic drift could impede adaptation to new environmental conditions by reducing standing adaptive genetic variability (Agashe, Falk, & Bolnick, 2011; Bridle & Vines, 2007; Swaegers et al., 2013). Additionally, insular populations are likely to be prevented from adapting by means of evolutionary rescue, in which immigrants promote adaptive evolution by introducing novel, beneficial alleles lost to drift, increasing population size and thus mutational opportunities, and reversing negative growth rates (Bell, 2013; Bell & Gonzalez, 2011).

Apart from their generally high susceptibility to extinction, island populations have long been a focus of study in the evolutionary literature due to their often unique contribution to the adaptive diversity of a species (Watanabe, Kazama, Omura, & Monaghan, 2014). For example, some characteristics appear to evolve faster than expected on islands because high standing adaptive genetic variation is believed to be the most influential mechanism for adaptation (Barrett & Schluter, 2008), and island populations generally exhibit low levels of genetic diversity (Nei et al., 1975). Such fast‐paced evolution on islands is likely determined by strong selective pressures for specific traits, driven by the highly divergent environments and community structures often encountered on island landscapes that are necessary to overwhelm the effects of genetic drift. This adaptive divergence is also likely reinforced by a lack of homogenizing gene flow with mainland populations. For example, Millien (2006) compared the rates of evolution between island and mainland populations of a number of mammal species and found that significant morphological changes can occur over short time intervals on islands. These changes are most commonly observed as differences in size (Lomolino, 1985, 2005; Lomolino et al., 2012, 2013), where smaller mammals on islands evolve toward gigantism (e.g., Millien & Damuth, 2004) and larger mammals evolve toward dwarfism (e.g., Roth, 1992; Sondaar, 1991). This graded trend, both across and within island taxa, was termed the “island rule” by Van Valen (1973) and has since been supported by numerous studies comparing species inhabiting both islands and the mainland (e.g., Dayan & Simberloff, 1998; Heaney, 1978; Kranzowski, 1967). The generality of the island rule is driven by the effect of body size on immigration potential, resource requirements, and inter‐ and intraspecific interactions of island species (Lomolino, 2005). Larger species, for example, experience a limitation in the amount of resources on islands and thus increased intraspecific competition, resulting in the higher fitness of smaller individuals who require less energy for survival and reproduction (Case, 1978; Lomolino, 1985, 2005). The validity of the island rule in regard to carnivores, however, has been challenged by some (i.e., Meiri, Dayan, & Simberloff, 2004, 2006), and the molecular mechanisms that underlie morphological differentiation are largely unknown.

As ecologically marginal and unique populations (e.g., edge or insular populations) are likely to encompass important components of genetic diversity, the loss of such populations are likely to result in the loss of rare and potentially beneficial adaptive genotypes (Watanabe et al., 2014). Such implications extend to management scenarios where human intervention is used as a means for the evolutionary rescue of island populations. If unique adaptations are present on islands, outbreeding depression may result from the introduction of maladapted individuals; the influx of their genes into the island population may swamp the well‐adapted gene pool and break down co‐adapted gene complexes (Miller, Poissant, Hogg, & Coltman, 2012). On the other hand, if genetic drift is predominating the distribution of island alleles and genotypes, the introduction of outside individuals may be an effective management strategy. Wide‐ranging species present unique challenges in this regard, due to high rates of gene flow and resulting genetic panmixia of the species across their range. For such species, insular populations may thus represent the only reservoirs of unique genetic material due to their isolation from the mainland, occupation of unique ecological settings, and exposure to divergent selection pressures (Gaston, 2003).

A classic example of a wide‐ranging and panmictic species is the Canada lynx (*Lynx canadensis*), which occurs throughout the boreal forests of Canada and Alaska, USA, and parts of the lower 48 United States. Canada lynx also inhabit the islands of Newfoundland and Cape Breton Island, Canada. Little is known, however, about the origin and subsequent isolation of these populations in comparison with the mainland population (Khidas, Duhaime, & Huynh, 2013). Row et al. (2012) conducted a genetic survey of lynx across their Canadian range, and found that lynx in Newfoundland were highly distinct at 21 neutral microsatellite markers. More recently, Koen, Bowman, and Wilson (2015) identified genetic differentiation between Canada lynx located north and south of the St. Lawrence River in Quebec, Canada. No such survey has been conducted for lynx on Cape Breton Island.

In addition to the inherent isolation of lynx on Cape Breton Island, morphological differentiation of Canada lynx across its range is greatest between individuals on Cape Breton Island and mainland Canada (Khidas et al., 2013). Specifically, of 18 craniodental traits measured by Khidas et al. (2013), both male and female lynx from Cape Breton Island had smaller and more variation in skull shape compared with the mainland population. This suggests that the body size of lynx on islands is smaller than in the mainland population, although this has not been explicitly tested. If consistent with the island rule, the smaller size of lynx on Cape Breton Island could be the result of their historically high population densities on the island (10–20 lynx/100 km^2^ during the peak of the population cycle; Parker, 1981, 2001), resulting in increased intraspecific competition for limited resources. A similar negative relationship between population density and body size has been reported for lynx in Alaska (Yom‐Tov et al., 2007). Further, evidence of differences in the social behavior (lynx on Cape Breton Island have been reported to regularly socialize in spruce bogs; Parker, 1981) and prey consumption (lynx on Cape Breton Island consume higher proportions of white‐tailed deer carrion or bait (Parker, Maxwell, Morton, & Smith, 1983) versus mainland lynx which rely on red squirrel as a secondary food source [O'Donoghue et al., 1998]) supports evolutionary differentiation between Cape Breton and mainland lynx populations.

Given the preceding evidence that lynx on Cape Breton Island have diverged phenotypically from individuals on the mainland, it is reasonable that both genetic drift and selection have contributed to their evolution and differentiation since their establishment on the Island, and perhaps on Newfoundland as well. Here, we used both neutral microsatellites and one non‐neutral (functional) genetic marker (insulin‐like growth factor‐1; *IGF‐1*) to evaluate the influence of genetic drift and natural selection on insular and mainland populations of Canada lynx in eastern Canada. First, we were interested in determining whether lynx on Cape Breton Island are genetically differentiated from lynx on mainland Canada. Genetic distinctness is likely for this population, given: (1) genetic differentiation at neutral molecular markers is proportional to time since divergence under an island model when it can be assumed that the population has descended from an ancestral population and since diverged without gene flow (Nielsen & Wakeley, 2001), and (2) lynx are thought to have occurred on Cape Breton Island as early as the 1600s (although these reports refer to Nova Scotia and not Cape Breton Island specifically; Denys, 1672). Second, as the function of the *IGF‐1* gene has been linked to body size in mammals (e.g., Sutter et al., 2007), we were interested in determining whether differentiation may be observable at the CA dinucleotide repeat between island and mainland lynx groups for which known morphological differentiation exists (Khidas et al., 2013). This repeat lies in the promoter region of the *IGF‐1* gene, containing regulatory elements that may alter transcription of the gene (Wagner et al., 2005). After Khidas et al. (2013), we hypothesized that lynx on Cape Breton Island are genetically divergent from mainland lynx, and predicted that we would observe high genetic distinction between lynx on Cape Breton Island and on the mainland at both neutral microsatellite markers and the non‐neutral *IGF‐1* dinucleotide repeat. Third, we were interested in assessing the contributions of genetic drift and natural selection to the observed allele frequencies of the *IGF‐1* locus in island versus mainland lynx. Lastly, we wanted to test whether genotypes at the *IGF‐1* locus were associated with measures of morphology in lynx on Cape Breton Island. If the gene is under selection or linked to a nearby locus under selection in lynx, we predicted that we would observe a relationship between common *IGF‐1* alleles found in lynx on Cape Breton Island and morphology.

## 2 Methods

### 2.1 Study area

**Figure 1:**
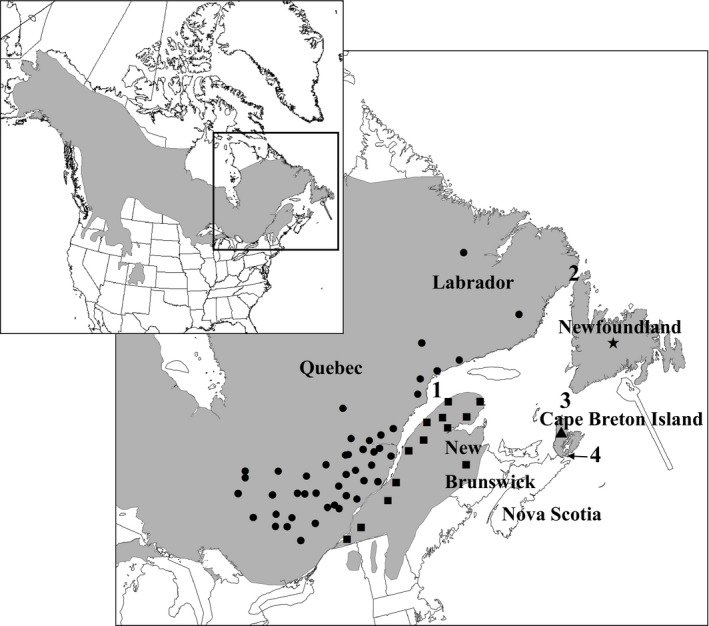
Sampling sites of 591 Canada lynx (*Lynx canadensis*) from eastern Canada. Samples from Quebec are spatially referenced to the centroid of harvesting units called Unités de gestion des animaux à fourrure (UGAF). Samples from Labrador, Newfoundland, and New Brunswick are plotted as the centroid of the province only. Samples from Cape Breton Island are plotted as the centroid of the Cape Breton Island highlands where lynx occur. Shapes represent the four genetic clusters identified by STRUCTURE analysis; Quebec north of the St. Lawrence River & Labrador (circles), Quebec south of the St. Lawrence River & New Brunswick (squares), Newfoundland (star), and Cape Breton Island (triangle). Isolating water channels are labeled numerically (1 = St. Lawrence River; 2 = Strait of Belle Isle, 17.6 km; 3 = Cabot Strait, 112 km; 4 = Strait of Canso, 1.6 km). Widths of the channels represent the narrowest point of each channel. The inset map identifies the study area within North America, and the distribution of Canada lynx (gray)

The mainland Nova Scotia population of Canada lynx was extirpated in the 1920s–1930s (Smith, 1940; de Vos & Matel, 1952), and now, the only remaining population of lynx in Nova Scotia occurs in the western plateau of Victoria and Inverness counties in the highlands of Cape Breton Island (Quinn & Parker, 1987; Figure 1). Cape Breton Island has a land area of approximately 9,500 km^2^ and is separated from mainland Nova Scotia by the Strait of Canso, which is approximately 1.6 km wide at its narrowest point (Matrin, 1837; Figure 1).

Historically, population densities of lynx on Cape Breton Island were high (Parker et al., 1983); however, more recent estimates of lynx density and area of occupancy found that only 50–500 animals remained on the island, depending on the stage of the population cycle (Parker, 2001). In 2002, Canada lynx were listed as endangered under the Nova Scotia Endangered Species Act and are now legally protected, such that harvesting of the remaining lynx on Cape Breton Island is prohibited (Nova Scotia Lynx Recovery Team 2006).

Although Cape Breton Island is isolated from mainland Nova Scotia, anecdotal evidence suggests that lynx move between Cape Breton Island and the mainland (the closest population being from New Brunswick), and it is speculated that lynx are using sea ice to traverse the Strait of Canso (Nova Scotia Lynx Recovery Team 2006). Uncertainty remains, however, as to whether migration to and from the island results in successful genetic exchange with mainland lynx (Poole, 2003).

### 2.2 Sample collection and DNA extraction

We sampled Canada lynx from Quebec (both north and south of the St. Lawrence River), New Brunswick, Labrador, and the islands of Newfoundland and Cape Breton (Figure 1). With the exception of Cape Breton Island, the samples we used in this study were the same as those in Koen et al. (2015), with some small changes in sample size. We collected hide samples from lynx that were harvested for their fur in Quebec (*N* = 493), Labrador (*N* = 21), and Newfoundland (*N* = 27) between 2008 and 2011. We obtained tissue samples from lynx that were incidentally harvested in New Brunswick (*N* = 15), in 2010. We obtained samples of Cape Breton Island lynx (*N* = 46) by extracting DNA from bone specimens that we obtained from the Canadian Museum of Nature in Ottawa, Ontario, Canada in 2013 (Supplement 1); these specimens were dated between 1977 and 1980.

### 2.3 Genetic profiling of samples

All lynx samples (excluding those obtained from Cape Breton Island) were amplified and genotyped at 14 presumably neutral microsatellite markers (*Fca031, Fca035, Fca043, Fca077, Fca090, Fca096, Fca441, Fca391, Fca559, Lc106, Lc109, Lc110, Lc111, and Lc118*) by Koen, Bowman, Lalor, and Wilson (2014a), according to the methodology described by Row et al. (2012). We followed the same protocol to amplify lynx samples from Cape Breton Island at these same 14 loci. We further amplified the dinucleotide microsatellite repeat within the *I*GF*‐1* functional gene in all samples with the primers described by Kirkpatrick (1992). For the *IGF‐*1 locus, we conducted amplification in a 10 μl reaction containing deionized water (Invitrogen), 1X PCR Reaction Buffer (Invitrogen), 2 mmol/L MgCl_2_ (Invitrogen), 0.2 mmol/L dNTP solution (Invitrogen), 0.2 mg/ml BSA, 0.4 μmol/L forward and reverse primers (forward primer labeled with the fluorescent dye HEX) (Integrated DNA Technologies), 0.025U Taq DNA Polymerase (Invitrogen), and 5 ng of DNA. We ran the PCR in a Bio‐Rad DNA Engine Dyad Disciple thermocycler under the following conditions: 95°C for 10 min followed by 30 cycles of 94°C for 30 s, 62°C for 1 min, and 72°C for 1 min, and completed with a step of 65°C for 15 min.

For genotyping of the *IGF‐1* locus, we mixed 5 μl of size standard (MapMarker 1000 X‐Rhodamine, BioVentures) into 1 ml of HiDi Formamide (Applied Biosystems), and we added 9.5 μl of this product to 0.5 μl of each amplified sample.

We performed genotyping on the Applied Biosystems 3730 DNA Analyzer. We scored genotypes for neutral microsatellites in lynx from Cape Breton Island and all lynx at the *IGF‐1* locus with Softgenetics LLC GeneMarker AFLP/Genotyping Software Version 1.91 (State College, PA, USA). We used manual scores from Koen et al. (2015) for all other samples. We omitted samples that were missing alleles at more than 2 of the 14 microsatellite loci. We analyzed the *IGF‐1* locus separately.

### 2.4 Genetic diversity and population structure

For analyses, we grouped samples into 6 sites based on their geographic location in eastern Canada; lynx sampled north of the St. Lawrence River in Quebec (*N* = 328), lynx sampled south of the St. Lawrence River in Quebec (*N* = 165), Labrador (*N* = 19), Newfoundland (*N* = 27), New Brunswick (*N* = 14), and Cape Breton Island (*N* = 38).

We used the R (R Core Team 2016) package adegenet, version 2.0.1 (Jombart, 2008) to estimate expected (*H*_E_) and observed (*H*_O_) heterozygosity for each locus, and conducted a *t*‐test to evaluate whether average expected and observed heterozygosity were significantly different across loci. We also used this package to conduct Bonferroni‐corrected (α = .05) chi‐squared tests to detect loci that departed from Hardy–Weinberg equilibrium (HWE). We used the software GENEPOP (version 4.2; Raymond & Rousset, 1995; Rousset, 2008) to conduct Bonferroni corrected (α = .05) tests for linkage disequilibrium (LD). We used HP‐RARE version 1.1. (Kalinowski, 2005) to estimate allelic richness (AR) for each of our 6 sites, and corrected our estimates with rarefaction for our lowest sample size (*N* = 14). We used the R package diversity (Keenan, McGinnity, Cross, Crozier, & Prodöhl, 2013) to obtain multiple estimates of genetic differentiation (*F*_ST_ [Weir & Cockerham, 1984]; *G*_ST_ [Nei, 1973]; and Jost's D [Jost, 2008]) as well as *F*_IS_, with 95% confidence intervals (CIs) on each estimate (999 bootstraps). We estimated AR, genetic differentiation, and *F*_IS_ separately for our set of 14 neutral microsatellites and the *IGF‐1* locus, due to the prospect that *IGF‐1* was under selection or linked to a nearby marker under selection. The *IGF‐1* data set consisted of lynx sampled from north of the St. Lawrence River in Quebec (*N* = 297), south of the St. Lawrence River in Quebec (*N* = 141), Labrador (*N* = 19), Newfoundland (*N* = 24), New Brunswick (*N* = 14), and Cape Breton Island (*N* = 38). All of these samples were from the same individuals that we sampled for neutral markers.

We used the Bayesian‐clustering software STRUCTURE version 2.3.4 (Pritchard, Stephens, & Donnelly, 2000) to identify genetic clusters based on our 14 neutral microsatellite loci (excluding the *IGF‐1* locus). We tested scenarios of *K* = 1–9 and ran 10 repetitions for each value of K. We used a burn‐in length of 500,000 Markov chain Monte Carlo iterations, followed by a run length of 1,000,000 iterations. We selected an admixture model without prior location information and used a correlated allele frequency model. We selected the most likely number of genetic clusters using the Evanno method (Evanno, Regnaut, & Goudet, 2005) implemented in the software STRUCTURE HARVESTER (Earl & vonHoldt, 2012). We summarized our 10 replicate runs for each value of K with the software CLUMPP (Jakobsson & Rosenberg, 2007), and visualized them with the software DISTRUCT (Rosenberg, 2004). To add support to our STRUCTURE results, we also estimated pairwise measures of *F*_ST_, *G*_ST_, and Jost's D between clusters identified in STRUCTURE, and implemented Fisher's exact tests to estimate whether genotype distributions at our 14 neutral microsatellites were significantly different between each pair of clusters (2000 MC replications, *p* < .001) with the R package diversity (Keenan et al., 2013). We assigned admixed individuals to the cluster that accounted for more than 50% of their ancestry.

### 2.5 Assessing the influence of genetic drift versus selection at the functional IGF‐1 locus

There are two alternative hypotheses explaining genotypic and allelic variation in insular lynx populations. First, allelic distributions may be largely influenced by genetic drift. If this were the case, we would expect to find evidence of small effective population sizes (*N*_e_), a history of population bottlenecks, and/or reduced genetic diversity across loci in island but not mainland populations. Alternatively, insular populations could be experiencing strong directional or positive selection, where few alleles are driven to high frequencies on the islands (not necessarily the same alleles as those at high frequency on the mainland) due to differences in environmental conditions, prey base and/or inter and intraspecific competition. In this case, we would expect values of genetic differentiation that are greater at the loci under selection (e.g., *IGF‐1*) in comparison with neutral markers (e.g., *F*_ST_ outliers), or a correlation of alleles with environmental or other variables that differ between the examined mainland and insular populations (e.g., morphology). To differentiate between these hypotheses, we conducted multiple analyzes.

To identify the contribution of genetic drift, we followed the methodology of Funk et al. (2016) using our neutral microsatellite marker dataset. First, to describe genetic diversity, we calculated three measures of within‐population genetic variation; observed (*H*_O_) and expected (*H*_E_) heterozygosity, and allelic richness (AR). We estimated these metrics using the same approaches as for our regionally defined populations (AR was corrected for our lowest sample size [*N* = 26] using rarefaction). We also estimated *N*_e_ for each of our genetic clusters using the molecular co‐ancestry method of Nomura (2008), as implemented in NeEstimator (version 2.01; Do et al., 2014). We used this method because it has been shown to provide unbiased estimates of *N*_e_, estimated as the effective number of breeders (*N*_eb_) for noninbred populations, and its narrower confidence intervals are more practical for interpretation (Nomura, 2008). Further, we estimated whether any of our genetic clusters have experienced recent bottlenecks using the software BOTTLENECK (version 1.2.02; Cornuet & Luikart, 1996). We used the default parameters under the two‐phase model (TPM) of mutation for our loci, as microsatellites with <3 bp repeats (e.g., our neutral dinucleotide repeats) generally have mutation models that include multiple‐step mutation events (Di Rienzo et al., 1994), and are therefore somewhere in between the standard step‐wise mutation model (SMM) and the infinite alleles model (IAM; Cornuet & Luikart, 1996). We ran 1,000 iterations for each test and used one‐tailed Wilcoxon signed rank tests to detect significant excess heterozygosity that is generally indicative of a bottleneck. Lastly, if genetic drift was influencing insular lynx, we would expect pairwise differentiation between genetic clusters to be negatively correlated with estimates of within‐population genetic variation (used as an index of historical genetic drift). Such an analysis has been used to identify genetic drift in populations of reptiles (Jordan & Snell, 2008), fish (Whiteley et al., 2010), and mammals (Funk et al., 2016). To test this hypothesis, we conducted linear regressions between pairwise estimates of genetic differentiation (*F*_ST_ and Jost's D) within our 4 genetic clusters, and average measures of within‐population genetic diversity (*H*_O_, *H*_E_, and AR) at our 14 neutral microsatellite markers. As gene flow with mainland populations is unlikely to have significantly contributed to within‐population genetic diversity of the islands (Funk et al., 2016), estimates of within‐population genetic diversity are an ideal index of historical genetic drift for this analysis.

We used the *IGF‐1* locus as an index of selection in our insular lynx populations. We plotted allele frequencies for our four genetic clusters (as identified with neutral markers by the program STRUCTURE; see section 3) at the *IGF‐1* locus to visualize the distribution of alleles, and used Fisher's exact tests implemented in GENEPOP (version 4.2; Raymond & Rousset, 1995; Rousset, 2008) to estimate whether allelic distributions were significantly different between each cluster. We also used a coalescent‐based (Beaumont & Nichols, 1996) outlier detection approach implemented in the software LOSITAN (Antao, Lopes, Lopes, Beja‐Pereora, & Luikart, 2008), which uses *F*_ST_ as a function of heterozygosity to identify loci under selection. We calculated the “neutral” mean *F*_ST_, where we first simulated removal of potentially selected loci prior to computing the initial mean *F*_ST_ upon which putative adaptive loci are identified. We also selected the option to “force mean *F*_ST_,” in which we approximated a more precise F_ST_ by running a bisection over repeated simulations. We ran 50,000 simulations at a 95% confidence interval, and selected a stepwise mutation model, which is commonly used to describe tandem repeat markers (Antao et al., 2008; Fan & Chu, 2007). Our LOSITAN tests were conducted using the genetic clusters identified with STRUCTURE. We ran an initial test across all four clusters, and then conducted tests removing each genetic cluster iteratively with replacement. In cases where LOSITAN identified a signature of selection at any locus, two additional independent tests were conducted as confirmation. We also plotted the pairwise Jost's D values of our neutral marker dataset with standard error of the mean intervals, and then overlaid each pairwise estimate of Jost's D at *IGF‐1* to determine whether these estimates fell within or outside of our expected range of neutrality. We used Jost's D here, as a complementary analysis to LOSITAN because *F*_ST_ and its analogs (e.g., *G*_ST_) are dependent on, and can be biased by within‐population heterozygosity, especially in cases when migration rates are expected to be low (Hedrick, 1999), whereas Jost's D uses the effective number of alleles (Kimura & Crow, 1964) which is a more appropriate measure for our outlier‐based approach.

### 2.6 Testing for associations between IGF‐1 alleles & morphology

As significant morphological divergence has been documented in insular lynx in comparison with mainland populations (Khidas et al., 2013), we were interested in testing whether these differences in morphology might be observable at a microsatellite repeat within the *IGF‐1* gene that has been linked to body size in some mammals (e.g., Baker, Liu, Robertson, & Efstratiadis, 1993; Sutter et al., 2007). Correlations between morphology and *IGF‐1* alleles would be indicative of the influence of selection on this locus in the same manner as the more conventional environmental association analyses (e.g., plants; Eckert et al., 2010; birds; Manthey & Moyle, 2015; and mammals; Ratkiewicz et al., 2014) and would provide evidence for a genetic basis of the island rule. First, we used Fisher's exact tests, implemented in the package diveRsity (Keenan et al., 2013), to estimate whether genotype distributions were significantly different for each pair of sites and each pair of clusters identified by STRUCTURE (2000 MC replications, *p* < .001).

We obtained seven morphological measures for each of our individual lynx genotyped on Cape Breton Island: total length, length of tail, length of hind foot, weight of skinned carcass, total length of skull, zygomatic width, and mandible length. We conducted 2‐way ANOVAs on each of these measured traits to determine whether mean trait values differed significantly by *IGF‐1* genotype, and corrected *p*‐values using Bonferroni correction (α = .05). Individuals with missing measurements were removed. We controlled for sex by including it as our second factor in each analysis, but were unable to control for age due to sample size limitations. Thus, to indirectly assess the influence of age on our measured traits, we estimated correlations between age and each trait using Pearson's correlation coefficients (for normally distributed data), or Spearman's rank correlation coefficients (for non‐normally distributed data).

## 3 Results

### 3.1 Genetic diversity and population structure

The number of alleles per locus ranged between 5 and 21, and mean expected and observed heterozygosity were significantly different (*p* < .001) across our set of 15 neutral and functional loci, suggesting deviations from HWE (Table S1). Specifically, Quebec lynx south of the St. Lawrence River departed from HWE at seven of our 15 loci (*IGF‐1*,* Lc106*,* Lc109, Lc110, Fca35, Fca96, and Fca441*;* p* < 3 × 10^−4^), whereas Quebec lynx north of the St. Lawrence River departed at only one locus (*Fca96*;* p* < 4 × 10^−5^). Insular lynx also showed deviations from HWE, with Newfoundland and Cape Breton Island deviating at 4 and 2 loci, respectively (Newfoundland: *Lc106, Lc111, Fca35, and Fca559*;* p* < 8 × 10^−5^; Cape Breton Island: *Lc118, Fca96*;* p* < 1 × 10^−4^). Lynx from Labrador and New Brunswick were within HWE at all loci. We identified five pairs of loci that showed evidence of LD (*IGF‐1* & *Fca391, Lc111* & *Lc118, Lc111* & *Fca31, Fca441* & *Fca31, and Fca441* & *Fca77*;* p* < .0004). Across all of our 14 neutral microsatellite loci, we detected only two sites (Quebec north and south of the St. Lawrence River) that significantly indicated inbreeding (95% CIs did not overlap 0), but the range of values of *F*_IS_ was small (0.005–0.038 and 0.023–0.079 for Quebec north and south of the St. Lawrence River, respectively), and thus inbreeding is likely not prevalent in these populations (Table S2). Allelic richness was lower in Newfoundland and Cape Breton Island in comparison with all mainland sites at both neutral markers (Newfoundland AR = 3.52; Cape Breton Island AR = 2.64) and the *IGF‐1* locus (Newfoundland AR = 2.00; Cape Breton Island AR = 2.00; Table S2). Private allelic richness, however, was lower in sites south of the St. Lawrence River (i.e., Quebec south of the St. Lawrence River, New Brunswick, and Cape Breton Island) than sites north of the river (i.e., Quebec north of the St. Lawrence River, Labrador, and Newfoundland) at both marker sets, with the exception that Newfoundland had a private allelic richness consistent with sites south of the St. Lawrence River at the *IGF‐1* locus (Table S2).

Estimates of genetic differentiation showed largely similar patterns for each of the metrics we measured (*F*_ST_, *G*_ST_ and Jost's D). Following Keenan et al. (2013), we plotted the relationship between mean number of alleles and each of our genetic differentiation metrics. The positive relationship that we observed between the mean number of alleles and both *F*_ST_ and *G*_ST_ indicated that high mutation rates were not biasing these estimates. Thus, we report genetic differentiation as *F*_ST_. As we predicted, the most highly differentiated sites were the islands (Table 1). The next most differentiated pairs of sites involved one of the two insular populations and one of the mainland sites, and differentiation was greater for comparisons including Newfoundland than Cape Breton Island (Table 1). Comparisons involving sites on the mainland were the most genetically similar, and, consistent with Koen et al. (2015), we found that sites north and south of the St. Lawrence River were more genetically differentiated than sites on the same side of the river. Overall, genetic differentiation between all pairs of sites was significant, with the exception of mainland sites on the same side of the St. Lawrence River (Quebec north of the river and Labrador, and Quebec south of the river and New Brunswick; Table 1). There was a slight change in the genetic structure of our sampled sites when genetic differentiation was measured at the *IGF‐1* locus (Table 1). The two insular populations were still the most differentiated sites; however, pairwise comparisons with Cape Breton Island were generally more highly differentiated at this locus than comparisons with Newfoundland. Evidence for higher genetic differentiation of mainland sites on opposite sides of the St. Lawrence River than sites on the same side of the river was also present at this locus.

**Table 1:** Pair‐wise *F*_ST_ (Weir & Cockerham, 1984) estimated at 14 neutral microsatellites (with 95% confidence intervals in parentheses; lower) and the *IGF‐*1 locus (upper), of 591 Canada lynx (*Lynx canadensis*) sampled in eastern Canada

	NFLD	LAB	QC_N	QC_S	NB	CBI
NFLD	–	0.2520	0.1397	0.0791	0.1578	0.2714
LAB	0.2004 (0.1541–0.2460)	–	0.0246	0.0943	0.0368	0.1925
QC_N	0.1681 (0.1430–0.1910)	0.0054 (−0.0062–0.0220)	–	0.0437	0.0347	0.1956
QC_S	0.2330 (0.2039–0.2588)	0.0515 (0.0312–0.0748)	0.0525 (0.0451–0.0600)	–	0.0067	0.2386
NB	0.2631 (0.2153–0.3137)	0.0495 (0.0200–0.0876)	0.0470 (0.0311–0.0683)	0.0062 (−0.0108–0.0314)	–	0.2707
CBI	0.4213 (0.3830–0.4581)	0.2185 (0.1876–0.2494)	0.1559 (0.1430–0.1692)	0.1659 (0.1520–0.1820)	0.2099 (0.1783–0.2476)	–

Sample sites are abbreviated and represent lynx from Quebec north of the St. Lawrence River (QC_N), Quebec south of the St. Lawrence River (QC_S), Labrador (LAB), New Brunswick (NB), and the islands of Newfoundland (NFLD) and Cape Breton (CBI)

**Figure 2:**
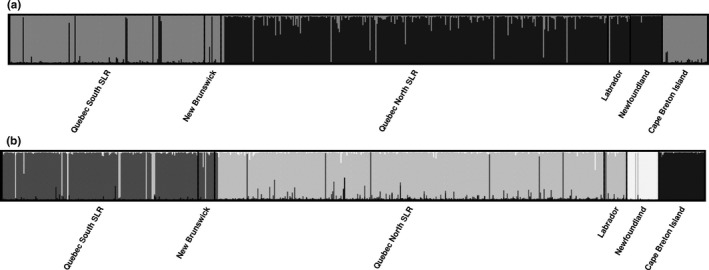
STRUCTURE plots of *K* = 2 (a) and *K* = 4 (b) representing the individual assignment values for 591 Canada lynx (*Lynx canadensis*) samples located in eastern Canada (based on shading). Plots are summaries of 10 replicates for each value of K

Both Evanno's Delta K and rate of change plots suggested two genetic clusters using our neutral microsatellite data set (Fig. S1A–C), separating sites north of the St. Lawrence River (Quebec north of the St. Lawrence River, Labrador, and Newfoundland) from sites south of the St. Lawrence River (Quebec south of the St. Lawrence River, New Brunswick, and Cape Breton Island; Figure 2a). Four genetic clusters were also identified with high support on these plots, however, separating Quebec north of the St. Lawrence River with Labrador, Quebec south of the St. Lawrence River with New Brunswick, Newfoundland, and Cape Breton Island (Figure 2b). Four genetic clusters were also identified as the most likely number of clusters in the Ln(K) plot (Fig. S1D). Estimates of genetic differentiation were consistent with the hypothesis that the Strait of Belle Isle (separating Newfoundland from the mainland), Strait of Canso (separating Cape Breton Island from the mainland), and the St. Lawrence River (separating northern from southern Quebec) are barriers to lynx gene flow (Table S3). We also found that the distribution of genotypes across our four genetic clusters were significantly different (*p* < .001) supporting four versus two genetic clusters. In concert with published evidence that Newfoundland (Row et al., 2012) and the St. Lawrence River (Koen et al., 2015) are impediments to gene flow for Canada lynx, we suggest that four genetic clusters are most likely for these data (Figure 1). We found that Newfoundland had greater differentiation at neutral markers and Cape Breton Island had greater differentiation at the *IGF‐1* locus whether we estimated genetic differentiation across our six sampling sites or four genetic clusters.

### 3.2 Assessing the influence of genetic drift versus selection at the IGF‐1 locus

**Figure 3:**
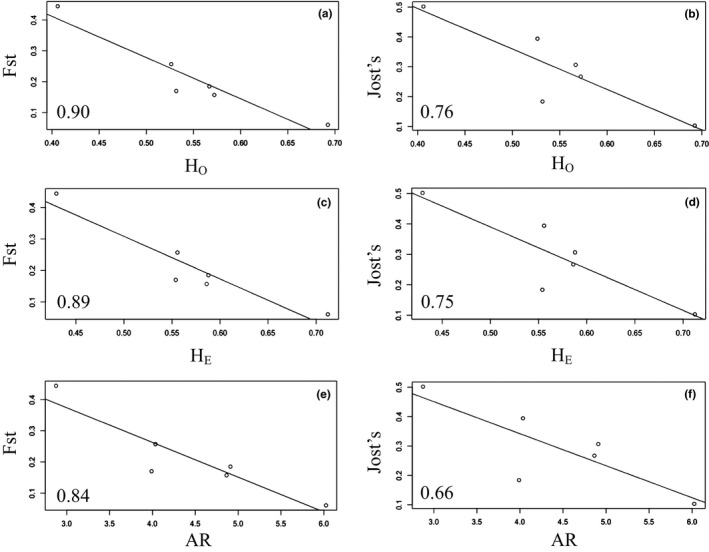
Linear regression plots of within‐population genetic diversity measured as observed heterozygosity (H_O_; a, b), expected heterozygosity (*H*_E_, c, d), and allelic richness (AR, e, f) with pairwise genetic differentiation measured as *F*_ST_ (a,c,e) and Jost's D (b, d, e) between insular and mainland genetic clusters of Canada lynx (*Lynx canadensis*) from eastern Canada. Multiple *R* ^2^ values are shown in the bottom left hand corner of each plot. All relationships were statistically significant (*F*_ST_ *p* < .01; Jost's D *p* < .05)

Estimates of genetic diversity within each of the four genetic clusters were lower for island clusters compared to mainland clusters at our 14 neutral microsatellite markers, as expected under the hypothesis of genetic drift (Table 2). Effective population sizes (*N*_e_) were small for the insular populations, whereas we estimated N_e_ of mainland sites as infinite, and thus likely to be large (Table 2). We detected evidence of bottlenecks in the form of heterozygosity excess for all four genetic clusters (*p* < .05), while there was slightly higher evidence for bottlenecks in the two mainland clusters (*p* < .01) than the island clusters (*p* < .05), there was also greater power in our analyses of the mainland sites as they had much larger sample sizes overall. Linear regressions between pairwise genetic differentiation and each of our within‐population measures of genetic diversity (*H*_O_, *H*_E_, and AR) were significant, regardless of the measure of genetic differentiation we used (*F*_ST_, *p* < .01; Jost's D, *p* < .05; Figure 3, Table 3), supporting the influence of drift in all lynx populations.

**Table 2:** Observed heterozygosity (*H*_O_), expected heterozygosity (*H*_E_), and allelic richness (AR) estimates calculated for 14 neutral microsatellite markers and the *IGF‐1* locus, and effective population size estimates (*N*_e_) with 95% confidence intervals (calculated from 14 neutral microsatellite loci) for 591 Canada lynx (*Lynx canadensis*) sampled from eastern Canada

Genetic cluster	*H*_O_ (neutral)	*H*_O_ (*IGF‐1*)	*H*_E_ (neutral)	*H*_E_ (*IGF‐1*)	AR (neutral)	AR (*IGF‐1*)	*N*_e_ (95% CI)
NFLD	0.40	0.26	0.43	0.29	2.92	2.00	6.5 (1.6–14.9)
QC_N & LAB	0.73	0.74	0.74	0.75	6.90	6.79	∞
QC_S & NB	0.65	0.49	0.68	0.56	5.15	4.75	∞
CBI	0.41	0.42	0.43	0.49	2.83	2.00	7.6 (2.1–16.7)

Sample sites are abbreviated and represent lynx from the four genetic clusters identified by STRUCTURE analysis; Quebec north of the St. Lawrence River with Labrador (QC_N & LAB), Quebec south of the St. Lawrence River with New Brunswick (QC_S & NB), and the islands of Newfoundland (NFLD), and Cape Breton (CBI)

**Figure 4:**
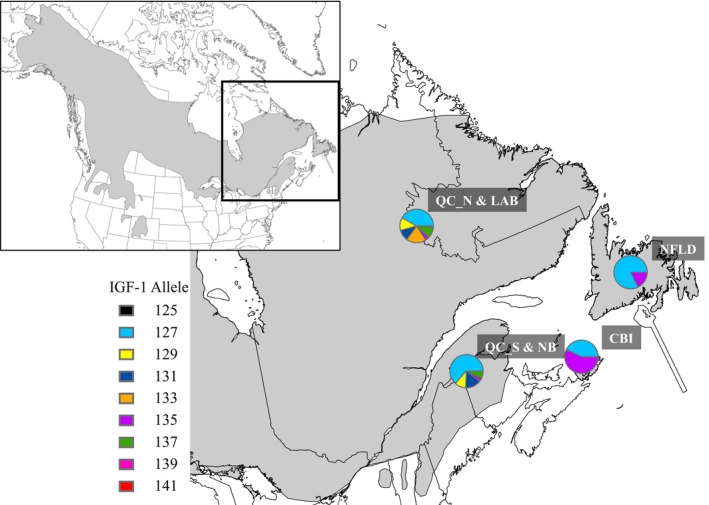
*IGF‐1* allele frequency distribution for 591 Canada lynx (*Lynx canadensis*) from eastern Canada. The inset map represents the study area within North America and the range of Canada lynx (gray). Sample sites are abbreviated and represent lynx from the four genetic clusters identified by STRUCTURE analysis; Quebec north of the St. Lawrence River with Labrador (QC_N & LAB; *N* = 316), Quebec south of the St. Lawrence River with New Brunswick (QC_S & NB; *N* = 155), and the islands of Newfoundland (NFLD; *N* = 24) and Cape Breton (CBI = 38)

Island populations of Canada lynx had lower genetic diversity than mainland clusters at the *IGF‐1* locus (Figure 4; Table 2). Allele frequencies at the *IGF‐1* locus were significantly different between all pairwise comparisons of the four genetic clusters (*p* < .001), and the most common allele found on Cape Breton Island (allele frequency of 0.569), which was also found at a relatively high frequency on Newfoundland (allele frequency of 0.174), was not found in any of the mainland clusters in high frequency (0.030–0.037 across mainland clusters; Table 4).

**Figure 5:**
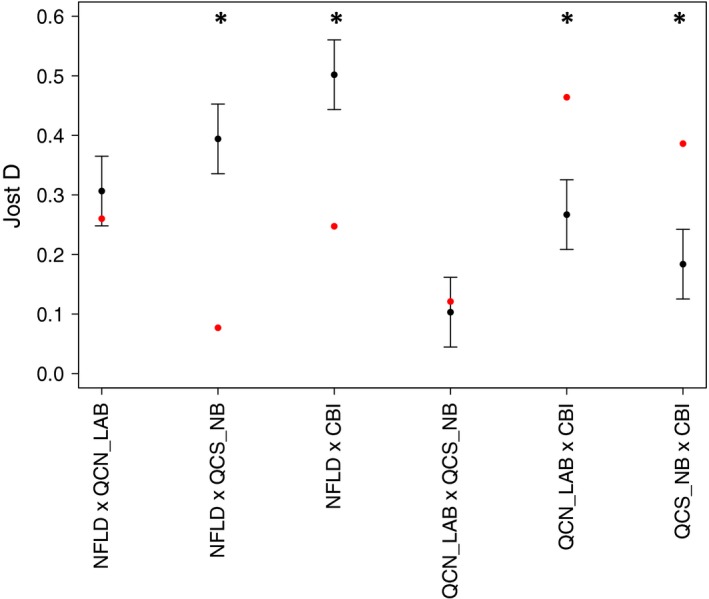
Pairwise point estimates of Jost's D for 14 neutral microsatellites (black dots) with bars representing standard error of the mean and point estimates of Jost's D for the *IGF‐1* locus (red dots) of 591 Canada lynx (*Lynx canadensis*) samples from eastern Canada. Significant results are indicated by asterisks at the top of the figure. Sample sites are abbreviated and represent lynx from the four genetic clusters identified by STRUCTURE analysis: Quebec north of the St. Lawrence River with Labrador (QCN_LAB), Quebec south of the St. Lawrence River with New Brunswick (QCS_NB), and the islands of Newfoundland (NFLD) and Cape Breton (CBI)

Our LOSITAN results provided no evidence that the *IGF‐1* locus was under positive selection. There was some evidence that microsatellite locus *Fca43* was under balancing selection, however, the locus consistently fell very close to the range of neutrality. In contrast to our LOSITAN results, our Jost's D plot comparing neutral microsatellites and *IGF‐1* indicated that the *IGF‐1* locus was potentially under selection in eastern populations of lynx. Specifically, genetic differentiation at the *IGF‐1* locus between the Cape Breton Island cluster and both of the mainland clusters fell outside of the standard error intervals estimated from the mean of our 14 neutral microsatellites (Figure 5). In addition, *IGF‐1* estimates of Jost's D fell outside of the standard error intervals when we compared Newfoundland to both Cape Breton Island and the Quebec south of the St. Lawrence River and New Brunswick clusters. All other estimates of Jost's D at the *IGF‐1* locus were within the standard error intervals generated from our neutral marker dataset (Figure 5). Further, estimates of Jost's D at the *IGF‐1* locus for all pairwise comparisons involving Newfoundland (including its comparison to Cape Breton Island) were lower than neutral expectations, whereas the mainland group comparison was approximately equivalent to neutral expectations, and pairwise comparisons with Cape Breton Island were higher than neutral expectations (Figure 5).

### 3.3 Testing for associations between IGF‐1 alleles and morphology

Our Fisher's exact tests indicated that the distribution of genotypes at the *IGF‐1* locus was significantly different across all pairs of sites (*p* < .05) excluding some comparisons involving two mainland sites (Labrador and New Brunswick, New Brunswick and Quebec north, and New Brunswick and Quebec south). When we conducted this estimation across genetic clusters identified using STRUCTURE, genotypic distributions were significantly different across all pairwise comparisons (*p* < .01).

All seven datasets used in our ANOVA analyses, had homogeneous variance. Sex was found to be a significant factor in two of our ANOVA analyses (total length, *F* = 14.75, *df* = 1, *p* = .0005; length of hind foot, *F* = 11.12, *df* = 1, *p* = .0022); however, the factor “*IGF‐1* genotype” was not significant for any of our traits following Bonferroni correction (Table S4). Age and trait variables were correlated for one data set only (zygomatic width, *t* = 2.508, *n* = 34, *p* = .0176).

## 4 Discussion

We found that lynx on Cape Breton Island were genetically distinct, supporting the hypothesis that the Strait of Canso is a barrier to gene flow for this insular population. Supported by previously published evidence of the Strait of Belle Isle isolating Newfoundland lynx (Row et al., 2012), and the St. Lawrence River isolating lynx north and south of the river (Koen et al., 2015), water barriers seem to be the most significant factor isolating peripheral populations of Canada lynx across their range. Patterns of genetic differentiation we report here were insensitive to the metric of genetic differentiation used (*F*_ST_, *G*_ST_ and Jost's D) and were consistent with expected patterns of gene flow for lynx (highest gene flow between mainland sites on the same side of the St. Lawrence River, and lowest between the island populations separated by the 112‐km wide Cabot Strait). Importantly, however, Cape Breton Island lynx were collected approximately 30 years (~10–15 generations) prior to all other samples, which could be contributing to the high levels of genetic differentiation reported here. Nonetheless, as the closest mainland population of lynx in Nova Scotia was extirpated long before the sampling of our Cape Breton lynx individuals, the substantial isolation (based on the Canso causeway and the considerable geographic distance to the nearest mainland population) of this population since the early 1900s supports high levels of contemporary genetic differentiation despite the gap in our sampling period.

Both genetic drift and natural selection seem to influence the genetic differentiation and diversity of insular lynx. Analyses with our neutral molecular markers showed evidence of (1) lower genetic diversity of insular populations in comparison with mainland groups; (2) lower effective population sizes of island versus mainland groups; (3) signatures of bottlenecks; and (4) negative relationships between pairwise genetic differentiation (*F*_ST_ and Jost's D) and within‐population genetic diversity (*H*_O_, *H*_E_, and AR). Combined, these results suggest that genetic drift has affected the genetic variation observed in island populations of Canada lynx. Interestingly, however, while AR of both neutral markers and the *IGF‐1* locus were lower in island versus mainland sites as predicted, private allelic richness of our neutral markers was lower in sites south of the St. Lawrence River, compared to sites north of the river. This result is consistent with the work of Koen, Bowman, Murray, and Wilson (2014b) who found that low private allelic richness of lynx in Ontario, Canada was associated with warm winter air temperature. It is possible that winter temperature is influencing this pattern as well, as winter temperatures tend to be lower south of the St. Lawrence River than in Newfoundland (Environment Canada, 2016).

We detected evidence of bottlenecks in our mainland genetic clusters at a higher level of significance than our island groups. We were expecting higher power in our analysis of the mainland groups; however, because the population sizes of our islands are small and thus bottlenecks are not likely to be as strong, reducing the period during which weak bottlenecks can be detected with high power (Cornuet & Luikart, 1996). In general, our BOTTLENECK analysis likely lacked power because the approach has lower power when loci evolve under a SMM (as opposed to the IAM) and have high heterozygosity (0.3 < H < 0.8) (Cornuet & Luikart, 1996). The bottlenecks detected in our mainland groups may merely reflect the difference in cyclic amplitude of Canada lynx (O'Donoghue et al., 1998; Stenseth et al., 1998; Yan, Stenseth, Krebs, & Zhang, 2013), which is lower or absent in peripheral populations of lynx (e.g., edge or island populations) in comparison with core populations (Murray, Steury, & Roth, 2008; Poole, 1994).

In addition to genetic drift, we were able to identify a functional locus that has likely been influenced by natural selection in insular lynx, *IGF‐1*, based on: (1) allele frequency differences; (2) contrasting patterns of genetic differentiation at neutral markers and *IGF‐1*; and (3) outlier tests using Jost's D. Combined, these results suggest that selection has also contributed to the genetic differentiation and diversity observed in island lynx populations. While it is true that the lower genetic diversity (e.g., allelic richness) of the *IGF‐1* locus on island compared to mainland groups of lynx could be the result of genetic drift, selection for fewer, but more favorable alleles on islands are better supported by our cumulative data. This is because both islands have retained the same two alleles despite likely being founded by genetically divergent populations on the mainland (north versus south of the St. Lawrence River). Further, the more common of the two alleles on Cape Breton Island, which is also found at a relatively high frequency in Newfoundland, was not found at high frequencies in either of the mainland sites of eastern Canada that we sampled, and there is likely a lack of gene flow between the two islands (supported by high estimates of *F*_ST_ and Jost's D). Thus, the unlikely prospect that an uncommon allele on the mainland would be present in the founding populations of both Cape Breton and Newfoundland and subsequently drifted to high frequencies on both islands lends support to the hypothesis that convergent selection has caused the increase in frequency of this allele in both populations of insular lynx simultaneously.

Patterns of differentiation also support a selection‐driven mechanism for the observed *IGF‐1* genotypes in our insular lynx populations. Specifically, while Newfoundland was more highly differentiated than Cape Breton Island with neutral genetic markers, the opposite trend was observed at the *IGF‐1* locus, suggesting that different forces are influencing genetic differentiation at these markers (i.e., drift vs. selection). Greater neutral genetic differentiation of Newfoundland is consistent with the premise that gene flow with the mainland is less prevalent in Newfoundland versus Cape Breton Island, which exchanges more migrants with the mainland via the Canso causeway (Nova Scotia Lynx Recovery Team 2006). At *IGF‐1*, however, Cape Breton Island was more highly differentiated, which could be attributed to selection occurring over a longer interval time on Cape Breton, as lynx are thought to have inhabited Cape Breton Island since at least the seventeenth century (Denys, 1672), whereas they were first reported on Newfoundland in 1897 (Bangs, 1897). Alternatively, there may be stronger selection on Cape Breton due to increased competition and predation (Parker, 2001; Parker et al., 1983; Vashon, Vashon, & Crowley, 2003), thereby limiting resources and selecting for the smaller body sizes observed on Cape Breton Island.

While we were unable to find evidence that the *IGF‐1* locus was under selection using the *F*_ST_ outlier detection approach, we were able to identify Cape Breton Island and Newfoundland as outliers using our Jost's D method in place of *F*_ST_. This is likely because Jost's D has been shown to perform better at estimating the differentiation in allele frequencies among populations, whereas *F*_ST_ is preferential for describing the effect of demography on genetic variation (Meirmans & Hedrick, 2011). In our analysis, Cape Breton and mainland group comparisons were significantly more differentiated at the *IGF‐1* locus than neutral expectations, indicating that divergent selective pressures are driving the differentiation of lynx on Cape Breton Island relative to mainland lynx at this locus. In contrast, lynx from Newfoundland compared to lynx from Cape Breton and Quebec south of the St. Lawrence River/New Brunswick were significantly less differentiated at the *IGF‐1* locus than expected from our neutral markers, indicating that similar selective pressures are decreasing differentiation between Newfoundland and other peripheral and isolated populations of lynx. Importantly, genetic differentiation of the mainland groups was very similar and nonsignificant between neutral markers and *IGF‐1*, suggesting that gene flow may overwhelm selection, and that selection on this locus is not strong enough to counteract this effect in these populations, resulting in patterns of differentiation at the *IGF‐1* locus that mimic neutral processes. Lastly, a nonsignificant deviation between *IGF‐1* and neutral marker estimates of Jost's D for Newfoundland, and its closest mainland relative, Quebec north of the St. Lawrence River/Labrador, suggests a strong effect of gene flow counteracting selection between these populations. This is more difficult to interpret; however, as gene flow is likely very limited between these two populations, which are separated by the Strait of Belle Isle, a minimum of 17.6 km wide barrier (Figure 1).

Although we did not find associations between morphological trait measurements and *IGF‐1* genotype of lynx on Cape Breton Island, there were limitations in our approach to detecting associations between genetic and morphological data. First, we cannot reject the possibility that unmeasured traits may be significantly associated with the *IGF‐1* genotypes observed on Cape Breton Island. Further, associations between morphology and the *IGF‐1* locus could be better understood by comparing across groups (e.g., on both islands as well as mainland groups), and increasing sample sizes within groups. Indeed, the probability of detecting significant associations between morphological traits and *IGF‐1* alleles would be greater if a larger variation of morphologically distinct phenotypes were compared across island and mainland sites; however, we did not have access to morphological measurements for the mainland lynx samples used in our study. Coupled with the fact that the Cape Breton Island population has low levels of variation in morphological trait measurements and high levels of gene flow, our analysis likely lacked power to associate common *IGF‐1* alleles with phenotype. Ultimately, addressing these limitations may allow for a better understanding of the genetic basis of the island rule in mammals. Taken together, our analyses of the *IGF‐1* locus indicated that while genetic drift may be strong in island populations, the strength of selection may also be strong enough to allow for the adaptive divergence of important traits, including body size.

Disentangling the contributions of genetic drift and natural selection in island populations is an important conservation goal with relevance to species management. The low genetic diversity of island Canada lynx populations via genetic drift reported here may suggest that lynx are limited in their capabilities to track changes in the environment, especially at a time‐scale that is consistent with climate change. Specifically, the ability of insular populations to adapt to environmental stressors is largely dependent on the population's standing adaptive genetic variation (Barrett & Schluter, 2008), which is essentially hindered in insular lynx due to their small effective population sizes and overall low levels of genetic diversity. This suggests that these island populations may rely largely on de novo mutations for adaptation, a mechanism that may not be very effective in short time frames. Consequently, the small, insular lynx populations of Newfoundland and Cape Breton Island are likely at higher risk of extirpation (see Frankham, 1997), and thus warrant appropriate conservation efforts.

Although extinction risk is more likely driven by genetic drift than selection in small populations (Whitlock, 2000), the identification of adaptively divergent traits further suggests that insular populations of Canada lynx warrant conservation efforts to preserve the unique alleles that are present at high frequencies on the islands but are largely absent from the mainland. Knowledge of adaptive genetic diversity may also benefit management plans by informing the potential for genetic rescue of these populations. A greater understanding of a larger breadth of adaptive genetic differences and/or similarities, however, is suggested if management actions based on adaptive genetic traits were under consideration. Thus, further work should seek to examine a wider variety of adaptive traits to more fully understand the adaptive landscape of both island and mainland lynx.

## 5 Conclusions

The protection of island populations is often a conservation goal due to their unique genetic contribution to the species. Integral to this goal is understanding the relative roles of genetic drift and natural selection in shaping the genetic diversity and differentiation of insular populations. The protection of adaptive genetic diversity within species is critical for ensuring species persistence in the face of both short‐term environmental stressors and long‐term climate change. Additionally, understanding the impact of genetic drift in isolated and often small island populations is a critical step toward understanding whether the loss of genetic variation imposes a significant threat to the long‐term persistence of populations via inbreeding depression. We showed that while genetic drift has played a role in shaping the observed genetic structure of insular Canada lynx populations, adaptive differentiation also seems to contribute to their unique gene pool. Extending the analysis of genes putatively under selection (Kirk & Freeland, 2011) will allow for a better understanding of the influence of selection on islands in traits that are not phenotypically apparent and will greatly contribute to our understanding of the genetic basis for selection in island environments. This could also translate to a better understanding of the effects of landscape fragmentation on the mainland, which essentially creates “island” populations in species with limited dispersal abilities.

## Author Contributions

Facilitated sample collection: JB, PJW, DM, and KK. Conceived and designed the experiments: MBP, JB, PJW, and KK. Performed the laboratory work and analyzed the data: MBP. Wrote the manuscript: MBP. Critically reviewed the manuscript: MBP, JB, KK, ELK, JR, DLM, and PJW.

## Conflict of interest

None declared.

## Data Accessibility

Data available from the Dryad Digital Repository: http://dx.doi.org/10.5061/dryad.8ff46.
