# Supplementary material for: Selection and drift influence genetic differentiation of insular Canada lynx (Lynx canadensis) on Newfoundland and Cape Breton Island
Source: Ecol Evol. 2017 Apr 9;7(9):3281–94. doi: 10.1002/ece3.2945 (PMC5415520; doi:10.1002/ece3.2945)
Supplement: Supplementary file 1 [file ECE3-7-3281-s001.docx]

**Selection and drift influence genetic differentiation of insular Canada lynx (*Lynx canadensis*) on Newfoundland and Cape Breton Island**

***Supplementary information, tables and figures***

Prentice MB, Bowman J, Khidas K, Koen EL, Row JR, Murray DL, Wilson PJ

DNA extraction from bone specimen:

We extracted bone specimens of Cape Breton lynx via a rapid column-based ancient DNA extraction protocol developed by Rohland et al. (2010). We collected inner nose bone tissue (250 mg) from each specimen and ground each sample using a freezer mill. Subsequently, we added 5mL of fresh extraction buffer (EDTA and Proteinase K), and incubated each sample under constant agitation for 16-24 hours in a dark room at room temperature. We centrifuged each sample for at least 2 minutes at 5,000g and transferred the supernatant into new tubes into which we added 2.5mL of binding buffer (GuSCN and sodium acetate) and 100ul of silica suspension (silicon dioxide and HCl). We incubated the samples under constant agitation for 3 hours in a dark room at room temperature, and then centrifuged them again for 2 minutes at 5,000g and discarded the supernatant. We added 400uL of binding buffer to each sample to re-suspend the silica pellet, and then transferred the suspension to a spin column in a collection tube. We centrifuged the samples for 30 seconds at 16,000g to pass the buffer through the silica and then discarded the buffer. We added 450ul of washing buffer (ethanol, NaCl, Tris and EDTA) to each sample, centrifuged the samples for 30 seconds at 16,000g, and discarded the buffer. We repeated the washing of the samples 1-2 times, depending on the colour of the sample (if the sample was still coloured, the steps were repeated). Finally, we added 50ul of 1x TE on the silica in the column, incubated the samples for 10 minutes at room temperature, and centrifuged each sample for 1 minute at 16,000g to produce the final product.

**Literature Cited:**

Rohland N, H Siedel, M Hofreiter (2010) A rapid column-based ancient DNA extraction method for increased sample throughput. Molecular Ecology Resources 10:677-683.

**Supplementary Table S1.**  Number of alleles, and observed and expected heterozygosity across sites for 14 neutral microsatellites and the *IGF-1* di-nucleotide repeat in Canada lynx (*Lynx canadensis*) from Quebec north of the St. Lawrence River (N= 328), Quebec south of the St. Lawrence Ricer (N= 165), New Brunswick (N= 14), Labrador (N= 19), Newfoundland (N= 27), and Cape Breton Island (N= 38). Canada. H_o_= observed heterozygosity, H_e_= expected heterozygosity.

| **Locus** | **Number of Alleles** | **Ho** | **He** |
| --- | --- | --- | --- |
| IGF-1 | 9 | 0.62 | 0.70 |
| Lc106 | 8 | 0.60 | 0.69 |
| Lc109 | 8 | 0.70 | 0.83 |
| Lc110 | 9 | 0.72 | 0.81 |
| Lc111 | 8 | 0.69 | 0.73 |
| Lc118 | 8 | 0.68 | 0.72 |
| Fca31 | 8 | 0.67 | 0.73 |
| Fca35 | 21 | 0.74 | 0.88 |
| Fca43 | 5 | 0.62 | 0.62 |
| Fca77 | 7 | 0.65 | 0.74 |
| Fca90 | 6 | 0.40 | 0.46 |
| Fca96 | 9 | 0.73 | 0.80 |
| Fca391 | 7 | 0.69 | 0.73 |
| Fca441 | 7 | 0.71 | 0.78 |
| Fca559 | 18 | 0.81 | 0.87 |

**Supplementary Table S2.**  Average allelic richness, private allelic richness, and inbreeding coefficients (F_IS_) with 95% confidence intervals (CI) for 591 Canada lynx (*Lynx canadensis*) across 14 neutral microsatellite loci. Also included is allelic richness estimates for the *IGF-1* locus (measured separately). Sample sites are abbreviated and represent lynx from Quebec north of the St. Lawrence River (QC_N; N= 328), Quebec south of the St. Lawrence River (QC_S; N= 165), Labrador (LAB= N= 19), New Brunswick (NB; N= 14), and the islands of Newfoundland (NFLD; N=27) and Cape Breton (CBI; N= 38), Canada.

| Sample Site | Allelic Richness (Neutral markers) | Allelic Richness (*IGF-1*) | Private Allelic Richness (Neutral markers) | Private Allelic Richness  (*IGF-1*) | F_IS_ | F_IS_ Upper CI (95%) | F_IS_ Lower CI (95%) |
| --- | --- | --- | --- | --- | --- | --- | --- |
| NFLD | 3.52 | 2.00 | 0.22 | 0.00 | 0.092 | -0.004 | 0.192 |
| LAB | 6.46 | 7.54 | 0.42 | 1.20 | -0.016 | -0.09 | 0.06 |
| QC_N | 6.08 | 6.07 | 0.29 | 0.24 | 0.021 | 0.005 | 0.038 |
| QC_S | 4.93 | 4.77 | 0.05 | 0.00 | 0.05 | 0.023 | 0.079 |
| NB | 4.98 | 4.00 | 0.16 | 0.00 | -0.015 | -0.132 | 0.088 |
| CBI | 2.64 | 2.00 | 0.01 | 0.00 | 0.041 | -0.045 | 0.129 |

**Supplementary Table S3.** Pair-wise F_ST_ (Weir & Cockerham 1984) estimated at 14 neutral microsatellites (with 95% confidence intervals in parentheses; lower) and the *IGF-*1 locus (upper), of 591 Canada lynx (*Lynx canadensis*) samples in eastern Canada. Sample sites are abbreviated and represent lynx from the 4 genetic clusters identified by STRUCTURE analysis; Quebec north of the St. Lawrence River with Labrador (QC_N & LAB), Quebec south of the St. Lawrence River with New Brunswick (QC_S & NB), and the islands of Newfoundland (NFLD) and Cape Breton (CBI).

|  | NFLD | QC_N & LAB | QC_S & NB | CBI |
| --- | --- | --- | --- | --- |
| NFLD | - | 0.1433 | 0.0739 | 0.2606 |
| QC_N & LAB | 0.1849 (0.1720 - 0.1991) | - | 0.0571 | 0.1918 |
| QC_S & NB | 0.2569 (0.2419 - 0.2728) | 0.0608 (0.0555 - 0.0663) | - | 0.2490 |
| CBI | 0.4442 (0.4198 - 0.4713) | 0.1572  (0.1449 - 0.1701) | 0.1701 (0.1560 - 0.1861) | - |

**Supplementary Table S4.** Sample size (N), average trait measurements, and standard error (SE) of Canada lynx (*Lynx canadensis*) from Cape Breton Island, Canada. For our ANOVA tests, trait was the dependent variable is the trait and the factors were gender (male or female) and genotype (“Homo127” were individuals homozygous for allele 127, “Homo135” were individuals homozygous for allele 135, and “Hetero” were individuals heterozygous for both alleles).

| **Trait** | **Factor 1 Level** | **Factor 2 Level** | **N** | **Average** | **SE** |
| --- | --- | --- | --- | --- | --- |
| Total Length (inches) | Females | Hetero | 9 | 85.78 | 0.56 |
|  |  | Homo127 | 5 | 90.10 | 0.93 |
|  |  | Homo135 | 8 | 85.94 | 1.46 |
|  | Males | Hetero | 7 | 89.79 | 1.03 |
|  |  | Homo127 | 3 | 90.67 | 1.20 |
|  |  | Homo135 | 7 | 90.57 | 1.00 |
| Length of tail (inches) | Females | Hetero | 9 | 10.11 | 0.14 |
|  |  | Homo127 | 5 | 11.30 | 0.54 |
|  |  | Homo135 | 8 | 10.75 | 0.25 |
|  | Males | Hetero | 7 | 10.36 | 0.42 |
|  |  | Homo127 | 3 | 10.17 | 0.33 |
|  |  | Homo135 | 7 | 12.54 | 1.25 |
| Length of hind foot (inches) | Females | Hetero | 8 | 19.63 | 0.16 |
|  |  | Homo127 | 4 | 19.50 | 0.29 |
|  |  | Homo135 | 8 | 19.75 | 0.56 |
|  | Males | Hetero | 5 | 20.30 | 0.37 |
|  |  | Homo127 | 3 | 21.00 | 0.58 |
|  |  | Homo135 | 8 | 21.06 | 0.37 |
| Weight of skinned carcass (lbs) | Females | Hetero | 9 | 12.05 | 0.74 |
|  |  | Homo127 | 5 | 15.80 | 0.54 |
|  |  | Homo135 | 8 | 14.97 | 1.09 |
|  | Males | Hetero | 7 | 15.26 | 0.74 |
|  |  | Homo127 | 3 | 16.67 | 1.61 |
|  |  | Homo135 | 8 | 14.53 | 0.81 |
| Total length of skull (inches) | Females | Hetero | 6 | 118.52 | 1.23 |
|  |  | Homo127 | 4 | 124.81 | 2.31 |
|  |  | Homo135 | 6 | 120.54 | 2.21 |
|  | Males | Hetero | 7 | 123.59 | 2.25 |
|  |  | Homo127 | 3 | 125.37 | 3.01 |
|  |  | Homo135 | 6 | 124.87 | 2.51 |
| Zygomatic width (mm) | Females | Hetero | 6 | 83.41 | 0.77 |
|  |  | Homo127 | 4 | 87.67 | 1.02 |
|  |  | Homo135 | 6 | 85.11 | 1.59 |
|  | Males | Hetero | 7 | 86.71 | 1.56 |
|  |  | Homo127 | 3 | 87.59 | 2.44 |
|  |  | Homo135 | 6 | 87.87 | 1.84 |
| Length of mandible (mm) | Females | Hetero | 6 | 76.52 | 0.89 |
|  |  | Homo127 | 4 | 80.29 | 1.12 |
|  |  | Homo135 | 6 | 77.34 | 1.47 |
|  | Males | Hetero | 7 | 80.10 | 1.29 |
|  |  | Homo127 | 3 | 80.44 | 1.98 |
|  |  | Homo135 | 6 | 80.34 | 1.87 |

**
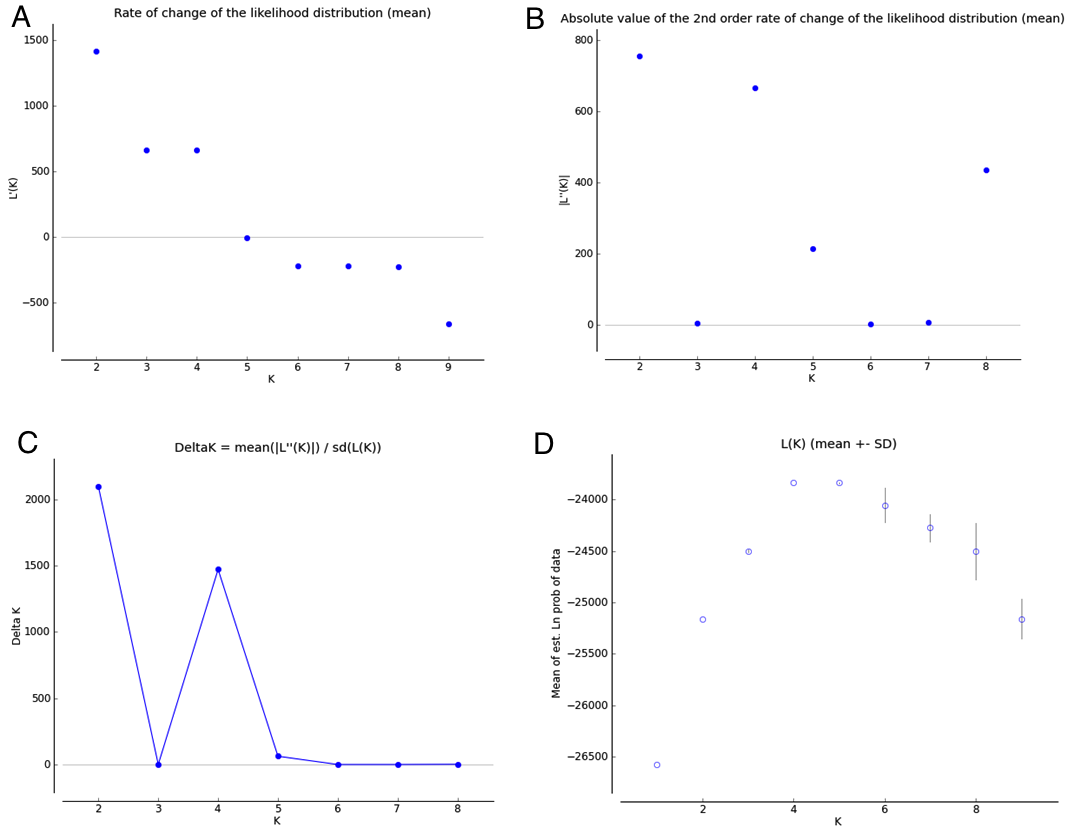
**

**Supplementary Figure S1.** Rate of change (A), absolute value of the 2^nd^ order rate of change (B), DeltaK (C), and LnK (D), plots produced by STRUCTURE HARVESTER (Earl & vanHoldt 2012) showing the likelihood for 1-10 genetic clusters of 591 mainland and insular Canada lynx (*Lynx canadensis*) from eastern Canada, genotyped at 14 neutral microsatellite loci.
